# Aerial Yam Bulbils Protect Against APAP-Induced Hepatotoxicity by Inhibiting Oxidative Stress and Mitochondrial Dysfunction Through Nrf2 Activation

**DOI:** 10.3390/nu17060966

**Published:** 2025-03-10

**Authors:** Siyu Xiang, Dong Kwon Yang

**Affiliations:** 1 Department of Veterinary Pharmacology & Toxicology, College of Veterinary Medicine, Jeonbuk National University, Specialized Campus, 79 Gobong-ro, Iksan 54596, Jeollabuk-do, Republic of Korea; xiangsiyu1023@naver.com; 2 Biosafety Research Institute and Laboratory of Pathology, College of Veterinary Medicine, Jeonbuk National University, Iksan 54596, Jeollabuk-do, Republic of Korea

**Keywords:** aerial yam bulbils, apoptosis, oxidative stress, MAPK, ER stress, Nrf2

## Abstract

Background/Objectives: The extract from aerial yam bulbils (AYB) contains various bioactive compounds, yet the mechanisms underlying its effects on APAP-induced liver injury need to be investigated further. This study sought to pursue the effects of AYB extract and the potential mechanisms involved in mitigating APAP-induced hepatotoxicity. Methods: TIB-73 cells were pretreated with AYB extract (10, 20, and 40 μg/mL) for 24 h and treated with APAP for 24 h to induce cytotoxicity. Results: Analysis of apoptosis-related proteins revealed that AYB extract exerts anti-apoptotic effects and inhibiting the MAPK signaling pathways, thereby reducing apoptotic cell death. Additionally, AYB extract significantly suppressed ROS overproduction by enhancing the expression of endogenous antioxidants and reducing the endoplasmic reticulum (ER) stress in APAP-treated cells, indicating that AYB extract inhibits APAP-induced oxidative stress. AYB extract effectively preserved mitochondrial membrane potential (MMP), maintained mitochondrial function-related genes, reduced mitochondrial oxidative stress, and mitigated mitochondrial damage, thereby preserving mitochondrial integrity. Additionally, AYB extract activated the Nrf2-related signaling pathway through nuclear translocation, leading to the upregulation of downstream antioxidative target genes. Diosgenin, a compound with known antioxidant properties and hepatoprotective effects, was identified in significant quantities in the AYB extract, suggesting that it may contribute to the observed hepatoprotective effects. Conclusions: Overall, these findings demonstrate that AYB extract, with its antioxidative properties, effectively protects TIB-73 cells from APAP-induced liver injury.

## 1. Introduction

Drug-induced liver injury refers to liver damage, both acute and chronic, caused by various medications, leading to abnormalities and dysfunction in liver tissues or cells [1]. Since it is a leading cause of acute liver failure in the US, accounting for 13% of acute liver failure cases, it presents a significant challenge for drug development and safety [2].

Acetaminophen (APAP), known as paracetamol, is widely used for its analgesic and antipyretic properties [3]. While APAP is generally safe and effective at therapeutic doses, overdose leads to drug-induced liver injury worldwide [4]. APAP metabolism via the Cytochrome P4502E (CYP4502E) enzyme produces *N*-acetyl-*p*-benzoquinone imine (NAPQI) that depletes glutathione and forms APAP-cysteine adducts [5]. In turn, this process leads to mitochondrial dysfunction, oxidative stress, hepatocyte necrosis, and liver injury [6].

Although hepatocyte apoptosis is a key pathological mechanism in liver diseases, with its extent and duration determining disease progression and outcomes, uncontrolled apoptosis results in extensive hepatocyte damage, inflammation, fibrosis, and, ultimately, liver failure or tumorigenesis in hepatic diseases [7]. Although APAP initially triggers multiple stress signals, particularly apoptotic process, hepatocytes exposed to APAP intoxication ultimately die through oncotic necrosis contributing to the loss of mitochondrial integrity, ATP depletion, and reactive oxygen species (ROS) formation [8].

Oxidative stress manifests when the body produces an excess of ROS, effectively overpowering its natural antioxidant defenses. [9]. The liver serves as the body’s key organ, expertly breaking down and detoxifying drugs, toxins, fats, and other substances [10]. These metabolic processes naturally generate ROS, which is involved in lipid metabolism and immune responses [11]. However, as a vital organ for oxidative detoxification, the liver faces significant levels of oxidative stress caused by external factors such as alcohol, toxins, heavy metals, and drugs, as well as internal conditions like obesity and diabetes [12]. Chronic oxidative stress activates hepatic stellate cells, prompting their differentiation into myofibroblasts, which accelerates liver fibrosis. This progression can lead to cirrhosis and, ultimately, liver failure [13].

Mitochondria play a pivotal role in various cellular processes, including the generation of ATP through lipid and carbohydrate metabolism, which fuels cellular activities [14]. Additionally, they act as key regulators of antioxidant defenses by balancing ROS production [15]. When mitochondrial function is impaired, excessive ROS accumulation leads to oxidative stress that further contributes to the development of liver diseases [16]. Furthermore, disruptions in mitochondrial homeostasis can impair signaling pathways related to mitochondrial-regulated lipid metabolism, inflammation, and hepatocyte apoptosis/necrosis. These disruptions can result in hepatocyte death, inflammation, and activation of hepatic stellate cells, ultimately driving fibrosis and cirrhosis [17]. Therefore, maintaining mitochondrial function is crucial for the treatment of liver diseases.

Aerial yam bulbils (AYB) of Chinese yam (*Dioscorea polystachya Turcz.*), which grow beneath the leaves of yam plants, are primarily used for sowing, propagation, and cultivation [18]. Previous studies indicated the immunomodulatory, anti-tumor, antioxidant, anti-aging, hypoglycemic, and lipid-lowering effects of Chinese yam (*Dioscorea opposita Thunb.*), highlighting the pharmacological potential of AYB as an emerging area of interest [19,20,21,22]. The primary constituents of AYB in Chinese yams (*Dioscorea bulbifera* L. and *Dioscorea opposita Thunb.*) include polysaccharides, proteins, flavonoids, and phenolic compounds [23,24]. Notably, polysaccharides of AYB in Chinese yam (*Dioscorea bulbifera* L.) exhibit significant free radical scavenging activity and possess potent anti-fatigue and antioxidant properties [23]. Additionally, AYB extract has been shown to effectively mitigate ethanol-induced hepatotoxicity due to its strong antioxidant activity [25].

Nuclear erythroid 2-related factor 2 (Nrf2) is a transcriptional factor for gene expression mediated by antioxidant response elements (ARE), playing an essential role in the transcriptional regulation of ARE-dependent defense genes [19]. In drug-induced liver injury, active metabolites can cause mitochondrial dysfunction and endoplasmic reticulum (ER) stress, triggering Nrf2 activation and the synthesis of antioxidant proteins to counteract cellular damage [26]. The Keap1-Nrf2 pathway protect mouse livers from lipotoxicity in non-alcoholic fatty liver disease [27]. Moreover, a previous study demonstrated that long-term treatment with CCl_4_ in Nrf2 knockout mice resulted in more severe inflammatory responses and increased liver fibrosis [28]. Under oxidative stress, Nrf2 dissociates from the inhibitory protein Keap1, accumulates, and translocates to the nucleus, where it binds to the ARE and activates the expression of various antioxidant genes, thereby providing cellular protection [29]. Additionally, numerous studies have shown that Nrf2 activation effectively reduces liver injury caused by APAP [19]. Thus, Nrf2 represents a critical target for the development of hepatoprotective drugs.

This study evaluated the potential of AYB extract to reverse APAP-induced liver injury in TIB-73 cells. The extract’s effectiveness in alleviating APAP-induced liver damage was assessed by examining its antioxidant, anti-apoptotic, and anti-mitochondrial dysfunction properties. These protective effects were mediated through the activation of the Nrf2 signaling pathway, involving Nrf2 translocation into the nucleus and subsequent upregulation of antioxidant gene expression.

## 2. Materials and Methods

### 2.1. AYB Extract

Yam bulbils were obtained from a Sarang nongwon (Jeonju, Republic of Korea). After thorough washing, the bulbils were dried in an oven at 60 °C, ground into powder, and extracted twice with 80% ethanol. The resulting solution was filtered, concentrated, and lyophilized. The extraction yield was 18.3%.

### 2.2. Cell Culture and AYB Extract, Silymarin and APAP Treatments

TIB-73 cells (ATCC; Manassas, VA, USA) were cultured in Dulbecco’s modified Eagle’s medium (DMEM; GIBCO-BRL, Grand Island, NE, USA) supplemented with 10% FBS and 1% antibiotics. The cells were maintained at 37 °C with 5% CO_2_. The AYB extracts were dissolved in dimethyl sulfoxide (DMSO; Sigma, St. Louis, MO, USA) to prepare stock solutions. For treatment, cells were pretreated with AYB extracts at final concentrations of 10, 20, and 40 μg/mL or 25 μg/mL silymarin (Sigma) for 24 h after dissolving with DMSO (Sigma, St. Louis, MO, USA), then treated with 10 mM APAP (Sigma) for an additional 24 h.

### 2.3. Cell Viability Assay

A 0.5 mg/mL MTT (Sigma) was incubated at 37 °C for 2 h, DMSO was added to dissolve formazan and measured the absorbance at 570 nm using a microplate reader (Spectra Max M5; Molecular Devices, Sunnyvale, CA, USA).

### 2.4. Hoechst 33342 Staining

Apoptotic cells were detected by nuclear staining using Hoechst 33342 dye (Thermo Fisher Scientific, Waltham, MA, USA). After fixed with 4% paraformaldehyde, 500 ng/mL Hoechst 33342 dye was added and incubated for 30 min at 37 °C. The nuclei were observed under a fluorescence microscope (Olympus Corp., Tokyo, Japan). The apoptotic index was calculated as the ratio of apoptotic cells to the total cells.

### 2.5. Western Blot Analysis

Proteins were extracted using RIPA (LPS Solution, Daejeon, Republic of Korea) with phosphatase and protease inhibitors. The lysates were then centrifuged, and the supernatant was collected. Proteins were separated by sodium dodecyl sulfate-polyacrylamide gel electrophoresis (SDS-PAGE) and transferred onto PVDF membranes. The membranes were blocked with 5% BSA for 1 h at RT. After blocking and incubated with primary antibodies for 6 h at 4 °C, followed by incubation with HRP-conjugated secondary antibodies for 1 h at RT. Proteins were detected using an enhanced chemiluminescence (Millipore Inc., Billerica, MA, USA) and visualized using a ChemiDoc imaging system (Cleaver Scientific Ltd., Warwickshire, UK). Details of the antibodies used for Western blot analysis are provided in Supplementary Table S1.

### 2.6. Measurement of Intracellular ROS

ROS levels were measured using DCFH-DA (Thermo Fisher Scientific Inc., Waltham, MA, USA). The cells were incubated with 1 μM DCFH-DA dye at 37 °C for 30 min. After incubation, fluorescence signals were detected using a fluorescence microscope with excitation at 488 nm and emission at 515 nm.

### 2.7. Measurement of Mitochondrial Membrane Potential

The mitochondrial membrane potential (MMP) was assessed using JC-1 (Enzo Biochem Inc., Farmingdale, NY, USA). An amount of 5 μg/mL JC-1 reagent was incubated at 37 °C for 10 min. Fluorescence signals were observed using a fluorescence microscope. Green fluorescence (indicating depolarized mitochondria) was detected at a 515/529 nm (excitation/emission wavelength), while red fluorescence (indicating polarized mitochondria) was detected at 485/590 nm (excitation/emission wavelength).

### 2.8. Measurement of Mitochondrial ROS Production

Mitochondrial ROS were detected using the MitoSOX Red mitochondrial superoxide indicator (Thermo Fisher). An amount of 5 μM MitoSOX reagent was incubated at 37 °C for 10 min. Images were captured using a live-cell imaging system (Oxford Instruments, Oxfordshire, UK).

### 2.9. Quantitative Real-Time Polymerase Chain Reaction (qRT-PCR)

Total RNA was isolated using the TRIzol reagent (Sigma). cDNA was synthesized using the GoScript™ RT System (Promega Co., Madison, WI, USA). Quantitative fluorescence analysis of target genes was performed using the TOPreal™ SYBR Green qPCR High ROX PreMIX kit (Enzynomics, Daejeon, Republic of Korea) on a real-time fluorescence qPCR instrument (Takara Bio Inc., Shiga, Japan). The primer sequences used for the qRT-PCR analysis are outlined in Supplementary Table S2.

### 2.10. Nuclear Fractionation of TIB-73 Cells

Nuclear fractions were obtained using a nuclear extraction kit (Abcam, Cambridge, UK). Cells treated with AYB extract and APAP were scraped into 1 mL of PBS, transferred to centrifuge tubes, and centrifuged at 1000 rpm for 5 min. Pre-extraction buffer was added to the pellet, incubated on ice for 10 min, and centrifuged at 12,000 rpm for 1 min. The supernatant containing the cytoplasmic extract was collected for Nrf2 Western blot analysis. To prepare the nuclear extract, a two-fold volume of extraction buffer containing DTT and phosphatase inhibitor was added to the nuclear pellet. The mixture was incubated on ice for 15 min and centrifuged at 14,000 rpm for 10 min. The resulting supernatant was collected for nuclear Nrf2 Western blot analysis.

### 2.11. Determination of Diosgenin Content

Diosgenin contents in the AYB extract solution were analyzed and quantified using an ultra-performance liquid chromatography-tandem mass spectrometry (UPLC-MS/MS) system (Waters Corp., Milford, MA, USA). The system was equipped with an ACQUITY UPLC BEH C8 column (2.1 mm × 100 mm, 1.7 µm; Waters Corp., Milford, MA, USA) maintained at 45 °C. The mobile phase consisted of water (solvent A) and acetonitrile (solvent B) at a flow rate of 0.45 mL/min, with an injection volume of 5 μL. Monitoring of the AYB extract was performed using the Waters XEVO TQ-S Micro series mass spectrometry system. The optimized mass spectrometry parameters were as follows: ion source voltage, 3.50 kV; source temperature, 150 °C; desolvation temperature, 350 °C; desolvation gas flow rate, 600 L/h; cone gas flow rate, 150 L/h. Data acquisition and processing were carried out using the MassLynx 4.1 software (Waters Corp., Milford, MA, USA). The diosgenin concentration in the AYB extract was calculated based on a standard curve.

### 2.12. Statistical Analysis

All results are expressed as the mean ± standard error of the mean (SEM). Statistical analyses were conducted using Prism 10.1.2 (GraphPad, San Diego, CA, USA). Statistical significance was determined via one-way analysis of variance (ANOVA) followed by Bonferroni post hoc tests. A *p*-value of less than 0.05 was considered statistically significant.

## 3. Results

### 3.1. AYB Extract Protects TIB-73 Cells from APAP-Induced Hepatotoxicity

**Figure 1:**
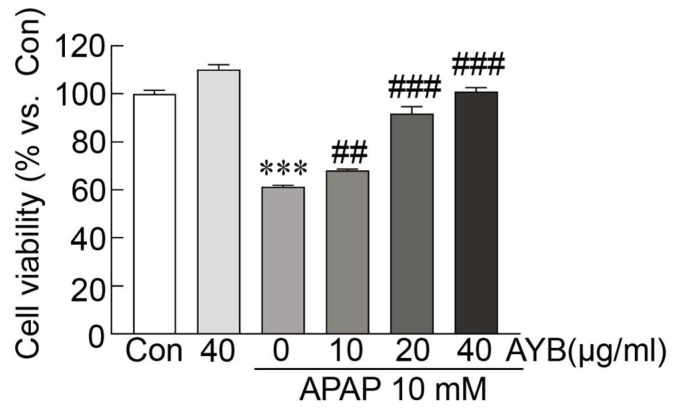
AYB extract inhibits APAP-induced hepatotoxicity in TIB-73 cells (n = 5). *** *p* < 0.001 vs. control. ## *p* < 0.01 and ### *p* <0.001 vs. APAP-alone-treated group. Con, control; AYB extract, aerial yam bulbils extract; APAP, acetaminophen.

To evaluate the protective effect of the AYB extract against APAP-induced hepatotoxicity, cell viability was measured using the MTT assay. TIB-73 cells treated with APAP, with or without AYB extract. Cell viability decreased to 61.27% upon treatment with APAP alone compared to controls (Figure 1). However, pretreatment with AYB extract enhanced cell viability in a dose-dependent manner with 68.25%, 91.80%, and 101.03% for the 10, 20, and 40 µg/mL AYB extract concentrations, respectively.

### 3.2. AYB Extract Inhibits TIB-73 Cells from APAP-Induced Apoptosis

**Figure 2:**
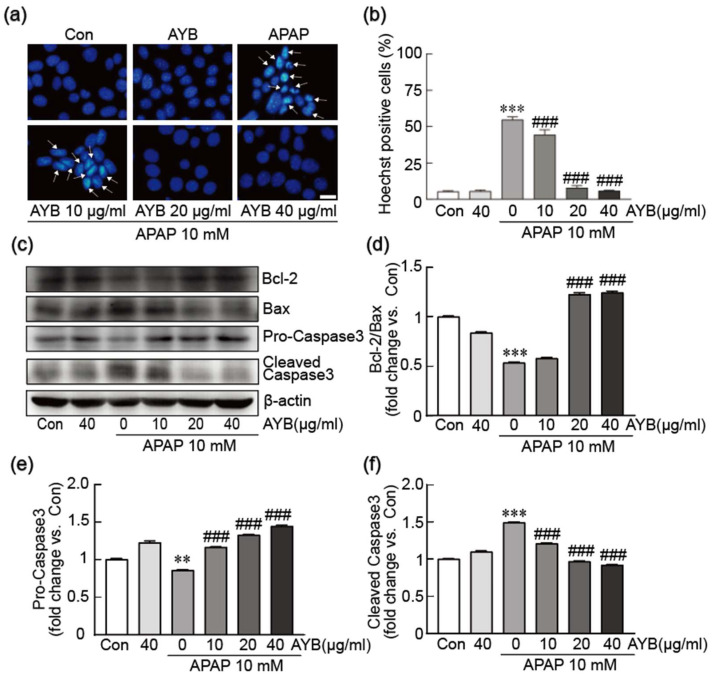
AYB extract inhibits APAP-induced apoptosis in TIB-73 cells. (**a**) Nuclear staining was performed using Hoechst staining. (**b**) The apoptotic index is shown as the percentage of apoptotic cells relative to total cells. (**c**–**f**) Western blot analysis of apoptosis-related proteins (n = 5). ** *p* < 0.01 and *** *p* < 0.001 vs. control. ### *p* <0.001 vs. APAP-alone-treated group. Con, control; AYB extract, aerial yam bulbils extract; APAP, acetaminophen. Scale bar, 100 µm. Arrow: apoptotic cells with nuclear condensation.

To investigate the inhibitory effects of AYB extract on APAP-induced apoptosis, nuclear condensation was assessed using Hoechst staining (Figure 2a,b). APAP-alone treatment led to an increase in nuclear condensation and apoptosis, affecting 54.73% of cells. In contrast, pretreatment with AYB extract at 20 and 40 µg/mL significantly reduced nuclear condensation, demonstrating a pronounced anti-apoptotic effect. The expression of key apoptosis-related proteins, including Bcl-2, Bax, and caspase-3, was further analyzed (Figure 2c–f). APAP treatment alone significantly reduced the expression of the anti-apoptotic protein Bcl-2, while increasing the pro-apoptotic protein Bax. In contrast, pretreatment with AYB extract restored Bcl-2 and Bax expression toward normal levels. Additionally, caspase-3 activation was assessed. APAP exposure led to a reduction in pro-caspase-3 (inactive form) and an increase in cleaved caspase-3 (active form), indicating the activation of this pro-apoptotic protein. In contrast, cells pretreated with AYB extract showed an elevation in pro-caspase-3 levels and a notable reduction in cleaved caspase-3, further supporting its protective and anti-apoptotic role.

### 3.3. AYB Extract Blocks APAP-Induced Oxidative Stress in TIB-73 Cells

**Figure 3:**
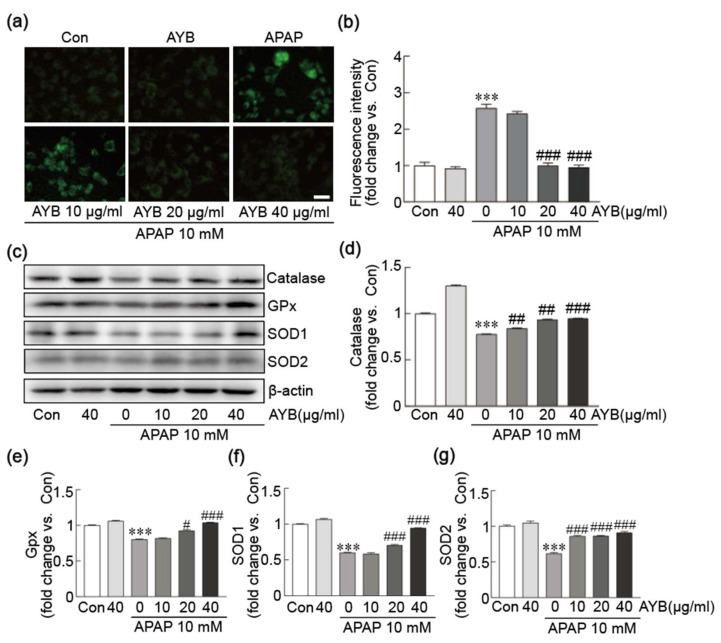
AYB extract inhibits TIB-73 cells from APAP-induced oxidative stress. (**a**) Green fluorescence intensity was measured using DCFH-DA staining. (**b**) Fluorescence intensity was quantified to visualize the experimental results. (**c**–**g**) Western blot analysis of oxidative stress-related proteins (n = 5). *** *p* < 0.001 vs. control. # *p* <0.05, ## *p* <0.01, and ### *p* <0.001 vs. APAP-alone-treated group. Con, control; AYB extract, aerial yam bulbils extract; APAP, acetaminophen. Scale bar, 100 µm.

The inhibitory effect of the AYB extract on APAP-induced oxidative stress was assessed by quantifying intracellular ROS levels using DCFH-DA dye (Figure 3a,b). APAP treatment alone significantly elevated ROS production in TIB-73 cells. In contrast, pretreatment with AYB extract at 20 and 40 µg/mL significantly reduced ROS levels, demonstrating its antioxidant potential. To further investigate the mechanism of ROS modulation, Western blot analysis was performed to assess the expression of key oxidative stress-related proteins, including catalase, GPX, SOD1, and SOD2 (Figure 3c–g). APAP exposure led to a substantial reduction in the expression of these antioxidant proteins. However, pretreatment with AYB extract significantly upregulated their levels, indicating its role in mitigating oxidative stress by enhancing the cellular antioxidant defense system.

### 3.4. AYB Extract Suppresses APAP-Induced Activation of the MAPK Signaling Pathway in TIB-73 Cells

**Figure 4:**
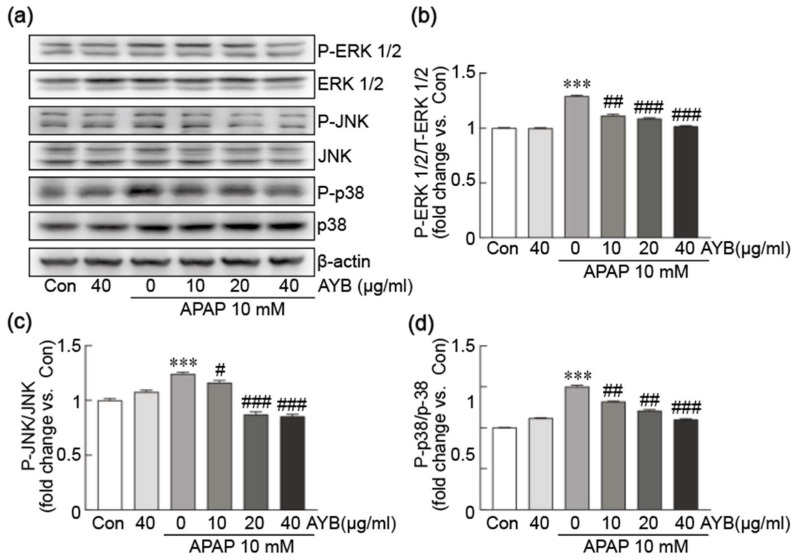
AYB extract inhibits APAP-induced activation of the MAPK signaling pathway in TIB-73 cells. (**a**–**d**) Western blot analysis of MAPK signaling pathway proteins (n = 5). *** *p* < 0.001 vs. control. # *p* <0.05, ## *p* <0.01, ### *p* <0.001 vs. APAP-alone-treated group. Con, control; AYB extract, aerial yam bulbils extract; APAP, acetaminophen.

The potential inhibitory effect of AYB extract on APAP-induced activation of the MAPK signaling pathway was evaluated by Western blot analysis, focusing on the canonical MAPK proteins ERK1/2, JNK, and p38 (Figure 4a–d). APAP treatment alone markedly increased the phosphorylation of ERK1/2, JNK, and p38 proteins, indicative of MAPK pathway activation. Pretreatment with AYB extract at 20 and 40 µg/mL significantly inhibited the phosphorylation of these proteins, suggesting that AYB extract effectively suppresses APAP-induced MAPK activation.

### 3.5. AYB Extract Mitigates APAP-Induced ER Stress in TIB-73 Cells

**Figure 5:**
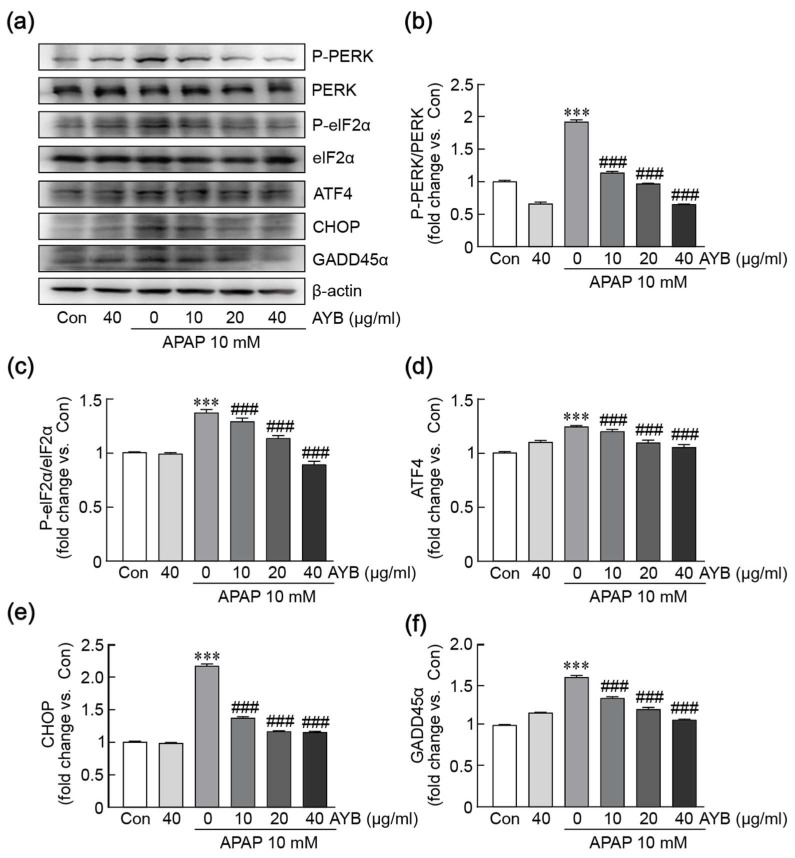
AYB extract inhibits APAP-induced ER stress in TIB-73 cells. (**a**–**f**) Western blot analysis of ER stress-related proteins (n = 5). *** *p* < 0.001 vs. control. ### *p* <0.001 vs. APAP-alone-treated group. Con, control; AYB extract, aerial yam bulbils extract; APAP, acetaminophen.

Oxidative stress and mitochondrial dysfunction are closely linked to ER stress. To explore this relationship, we analyzed the expression of ER stress-related proteins, including PERK, eIF2α, ATF4, CHOP, and GADD45α, via Western blot analysis (Figure 5a–f). Treatment with APAP alone significantly increased the phosphorylation of PERK and eIF2α, accompanied by elevated expression levels of ATF4, CHOP, and GADD45α proteins. However, pretreatment with AYB extract notably reduced the phosphorylation of PERK and eIF2α, and the expression levels of ATF4, CHOP, and GADD45α, indicating that APAP induces ER stress activation, while AYB extract effectively suppresses this activation.

### 3.6. AYB Extract Protects Against APAP-Induced Mitochondrial Dysfunction in TIB-73 Cells

**Figure 6:**
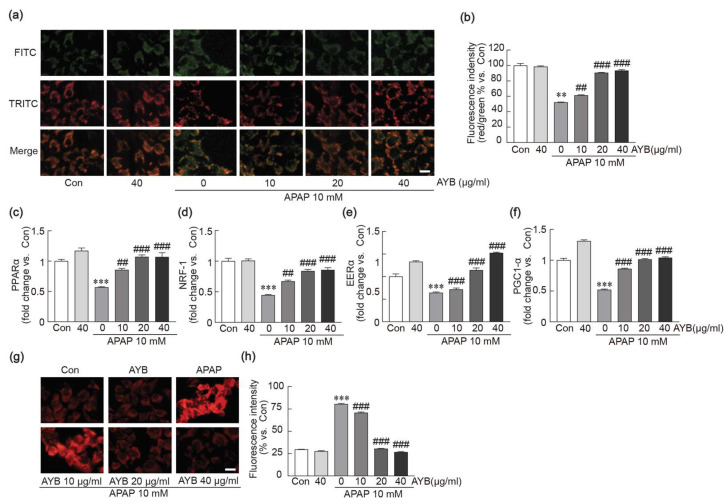
AYB extract inhibits APAP-induced mitochondrial dysfunction in TIB-73 cells. (**a**,**b**) The intensities of green and red fluorescence were measured using JC-1 staining. (**c**–**f**) Mitochondrial functional genes were analyzed by qRT-PCR in triplicate. (**g**,**h**) The intensity of red fluorescence was measured using MitoSOX staining. ** *p* < 0.01, *** *p* < 0.001 vs. control. ## *p* <0.01, ### *p* <0.001 vs. APAP-alone-treated group. Con, control; AYB extract, aerial yam bulbils extract; APAP, acetaminophen. Scale bar, 100 µm.

The protective effects of AYB extract against APAP-induced mitochondrial dysfunction were evaluated by observing mitochondrial membrane potential using JC-1 dye (Figure 6a,b). APAP treatment alone significantly decreased the red-to-green fluorescence ratio, reflecting a loss of mitochondrial membrane potential. In contrast, pretreatment with AYB extract significantly restored this ratio, indicating protection against mitochondrial membrane potential imbalance. Additionally, mitochondrial oxidative stress was assessed using the MitoSOX kit to label mitochondrial ROS (Figure 6g,h). APAP treatment alone caused a marked increase in red fluorescence intensity, indicating elevated mitochondrial ROS levels. In contrast, pretreatment with AYB extract effectively reduced ROS levels, demonstrating its ability to mitigate APAP-induced mitochondrial oxidative stress. Moreover, qRT-PCR analysis was conducted to evaluate the expression of key mitochondrial functional genes, including PPARα, NRF-1, ERRα, and PGC1-α (Figure 6c–f). APAP treatment alone significantly suppressed the expression of these genes. However, pretreatment with AYB extract markedly restored their expression, highlighting its role in maintaining mitochondrial function.

### 3.7. AYB Extract Mitigates APAP-Induced Oxidative Stress in TIB-73 Cells via Activation of the Nrf2 Pathway

**Figure 7:**
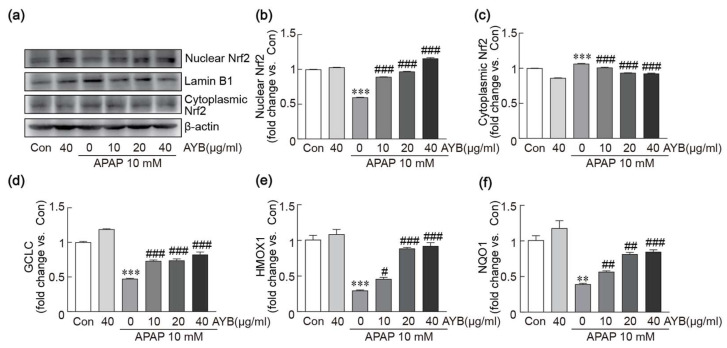
AYB extract inhibits APAP-induced oxidative stress in TIB-73 cells by activating the Nrf2 pathway. (**a**–**c**) Western blot analysis of nuclear and cytoplasmic Nrf2 (Lamin B1as a nuclear loading control and β-actin as a cytoplasmic loading control, n = 5). (**d**–**f**) The mRNA expression levels of Nrf2 downstream genes were analyzed by qRT-PCR in triplicate. ** *p* < 0.01, *** *p* < 0.001 vs. control. # *p* <0.05, ## *p* <0.01, ### *p* <0.001 vs. APAP-alone-treated group. Con, control; AYB extract, aerial yam bulbils extract; APAP, acetaminophen.

To explore the mechanism of AYB extract in APAP-induced liver toxicity, we investigated the activation of the Nrf2 signaling pathway as a central to cellular antioxidant defense. Western blot analysis revealed a significant reduction in nuclear Nrf2 levels and an increase in cytoplasmic Nrf2 levels in cells treated with APAP alone. In contrast, pretreatment with AYB extract restored nuclear and cytoplasmic Nrf2 protein levels to baseline levels in TIB-73 cells (Figure 7a–c), indicating that AYB extract activates Nrf2. Additionally, quantitative RT-PCR analysis of downstream genes within the Nrf2 signaling pathway, including GCLC, HMOX1, and NQO1, revealed a marked suppression of their expression in cells treated with APAP alone. However, pretreatment with AYB extract significantly restored the expression of these genes (Figure 7d–f). These findings suggest that AYB extract alleviates APAP-induced oxidative stress by activating the Nrf2 pathway and its associated downstream antioxidant responses.

### 3.8. UPLC-MS Analysis of Diosgenin Content in AYB Extracts

**Figure 8:**
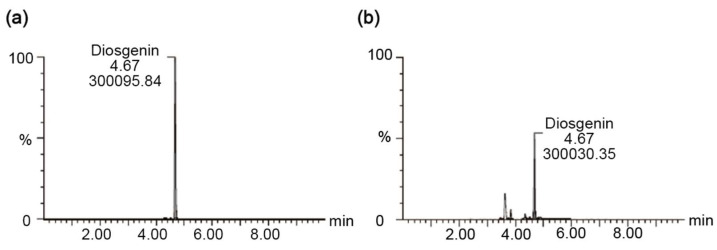
UPLC-MS analysis of diosgenin in the AYB extract. Representative UPLC-MS chromatogram of the diosgenin standard solution (**a**) and the diosgenin content in the AYB extract (**b**). AYB extract, aerial yam bulbils extract.

For the UPLC-MS analyses, the retention time of diosgenin was identified based on its retention time, which was matched to that of a diosgenin standard. The analysis determined that the diosgenin content in AYB extract was 977 ng/mL (Figure 8).

### 3.9. The Protective Effect of AYB Extract Is Similar to Those of Silymarin on APAP-Induced Hepatotoxicity

**Figure 9:**
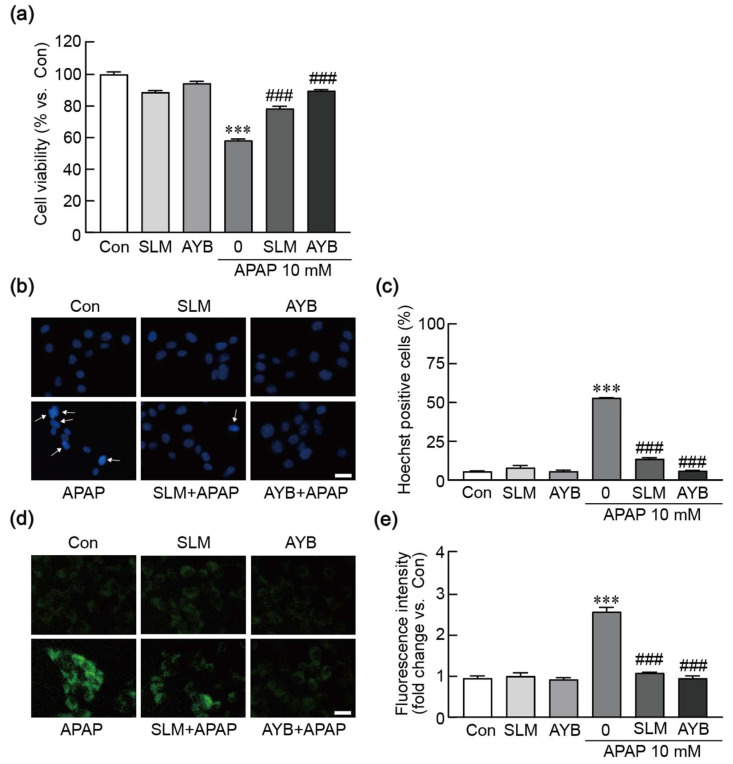
Comparison of the effects of AYB extract and silymarin on APAP-induced hepatotoxicity (n = 5). (**a**) Cell viability assay. (**b**) Nuclear staining was performed using Hoechst staining. (**c**) The apoptotic index is shown as the percentage of apoptotic cells relative to total cells. (**d**) Green fluorescence intensity was measured using DCFH-DA staining. (**e**) Fluorescence intensity was quantified to visualize the experimental results. *** *p* < 0.001 vs. control. ### *p* <0.001 vs. APAP-alone-treated group. Con, control; SLM, silymarin; AYB extract, aerial yam bulbils extract; APAP, acetaminophen. Scale bar, 100 µm. Arrow: apoptotic cells with nuclear condensation.

We conducted a study to evaluate the protective effects of AYB extract by comparing them with the protective effects of silymarin, which is well-known for its ability to protect against liver diseases, particularly APAP-induced hepatotoxicity [30]. The cell viability assay showed that pretreatment with 25 µg/mL silymarin and 40 µg/mL AYB extract increased the viability of TIB-73 cells to 70.84% and 89.72%, compared to control, respectively (Figure 9a). Hoechst staining indicated that pretreatment with silymarin and AYB extract significantly reduced nuclear condensation, with AYB extract exhibiting a more pronounced anti-apoptotic effect (Figure 9b,c). Furthermore, DCFH-DA staining demonstrated that pretreatment with 25 µg/mL SLM and 40 µg/mL AYB extract markedly reduced ROS levels, with AYB extract displaying a more potent antioxidant effect (Figure 9d,e). Overall, the protective effects of AYB extract are similar to those of silymarin on APAP-induced hepatotoxicity.

## 4. Discussion

Yam extract has been shown to possess hepatoprotective effects, which has been linked to its antioxidant activity [31]. However, studies on the protective effects of AYB extract remain limited. Our previous research demonstrated that AYB extract alleviates ethanol-induced hepatotoxicity by inhibiting oxidative stress [25]. This study aims to investigate whether AYB extract exhibits protective effects against APAP-induced liver injury.

To assess this, we conducted a cell viability assay to determine whether the AYB extract could restore cell viability. The results demonstrated its strong protective capacity against cellular damage. Hepatocyte apoptosis is a major cause of liver injury, involving both intrinsic and extrinsic pathways of programmed cell death, which activate executioner caspase-3. This leads to proteolysis, nuclear fragmentation, and apoptosis [32]. AYB extract successfully restored cell viability and DNA integrity compromised by APAP and inhibited the activation of cleaved caspase-3. Regarding anti-apoptotic protein expression, AYB extract exerted anti-apoptotic effects by upregulating Bcl-2 expression, thereby inhibiting the activation of the Bax protein [33].

APAP-induced hepatotoxicity is characterized by extensive oxidative stress. One pathway through which APAP generates ROS is via its metabolism by CYP450 to form NAPQI, which depletes glutathione (GSH) and leads to lipid peroxide accumulation, generating ROS [34]. Another pathway involves NAPQI forming protein adducts within mitochondria, thereby triggering oxidative stress [35]. Intracellularly, catalase, SOD1, SOD2, and GPx play crucial roles in maintaining mitochondrial homeostasis and eliminating ROS [36]. SOD1 and SOD2 convert superoxide anions, located in the mitochondrial intermembrane space and matrix, respectively, into H_2_O_2_, which is further broken down by catalase and GPx [37]. This reduces ROS-induced damage to mitochondrial proteins, lipids, and DNA, thereby regulating oxidative stress levels across the cell and mitigating mitochondrial stress [38]. In our study, AYB effectively reversed the APAP-induced downregulation of the antioxidant enzymes catalase, SOD, and GPx, in addition to inhibiting intracellular ROS production.

MMP and mitochondrial oxidative stress are closely interconnected [39]. When MMP is elevated, electron transport efficiency decreases, causing electrons to leak at complexes I and III, generating superoxide anions and other ROS [40]. The accumulation of ROS further induces oxidative stress, damaging the mitochondrial membrane and leading to a decline in MMP [41]. This decrease in MMP disrupts normal respiratory chain function, resulting in additional electron leakage and further ROS production. This creates a vicious cycle that exacerbates oxidative stress, with sustained ROS exposure causing further structural and functional damage to mitochondria, ultimately leading to irreversible mitochondrial impairment. In our experiments, we observed mitochondrial membrane potential using JC-1 staining and found that AYB extract could restore the APAP-induced MMP imbalance and the increase in mitochondrial ROS. We also analyzed proteins involved in mitochondrial biogenesis, energy metabolism, and antioxidant functions, and found that the AYB extract helped maintain mitochondrial homeostasis and reduce oxidative stress by upregulating the expression of PPARα, NRF-1, ERRα, and PGC-1α.

Prolonged or excessive oxidative stress can lead to protein oxidation and mitochondrial dysfunction, which increases the production of misfolded proteins and triggers ER stress [42]. Upon ER stress, the unfolded protein response (UPR) is activated for restoring ER homeostasis and reducing misfolded protein accumulation [43]. Failure of the UPR to effectively restore balance activates the MAPK signaling pathway, specifically JNK and p38, pushing the cell toward apoptosis [44]. Upon activation, JNK phosphorylates several apoptosis-related proteins, such as p53 and Bax from the Bcl-2 family, inducing changes in mitochondrial outer membrane permeability and further triggering apoptosis through the mitochondrial pathway [45]. The MAPK pathway links cellular responses to oxidative stress and ER stress by regulating ROS generation, ER stress induction, and apoptosis [46]. PERK is a key regulator in the ER stress response. Under ER stress conditions, Activated PERK triggers eIF2α, which promotes ATF4 expression, subsequently upregulating CHOP as a pro-apoptotic protein and accelerating the process of cell death [47]. GADD45α, regulated by CHOP, plays an essential role in DNA damage response, oxidative stress, and the cell cycle [48]. Collectively, our findings indicated that AYB extract administration reduces APAP-induced ER stress.

Nrf2 interacts with several antioxidant genes, including GCLC, HMOX1, and NQO1, playing an important role in maintaining redox balance and responding to cellular stress [49]. GCLC is the rate-limiting enzyme in GSH synthesis. When Nrf2 is activated, it upregulates GCLC expression, promoting GSH synthesis, clearing ROS, and maintaining intracellular redox balance [50]. HMOX1 is responsible for heme degradation, converting heme into biliverdin, carbon monoxide, and iron, with biliverdin and its subsequent products exhibiting strong antioxidant properties [51]. NQO1 is another Nrf2-regulated antioxidant enzyme gene that reduces quinones to non-radical forms, thus decreasing quinone-induced oxidative damage to cells [52]. By regulating NQO1 expression, Nrf2 aids in the clearance of oxidative products, protecting cells from oxidative stress. Overall, Nrf2 plays a significant role in maintaining cellular redox homeostasis by regulating antioxidant genes such as GCLC, HMOX1, and NQO1. Nuclear proteins were extracted to investigate whether Nrf2 promotes antioxidant gene expression through nuclear translocation. Our findings revealed that AYB extract activates Nrf2, enhancing antioxidant gene expression and reducing APAP-induced damage.

Our study demonstrates that AYB extract exerts hepatoprotective effects by promoting Nrf2 nuclear translocation, activating downstream antioxidant genes, inhibiting mitochondrial stress and dysfunction, and alleviating intracellular oxidative and ER stress. Given the complex mixture of compounds in AYB extract, future studies will focus on identifying its active components. In a previous study, diosgenin from yam extract demonstrated significant antioxidant and anti-inflammatory effects in a carbon tetrachloride-induced liver injury model, effectively reducing liver damage [53]. Additionally, dioscin, a precursor compound, has shown protective effects against APAP-induced hepatotoxicity [54]. Therefore, we measured the diosgenin content in AYB extract and found significant quantities, suggesting that diosgenin may, at least partially, contribute to its hepatoprotective effects through its antioxidant activity.

We further evaluate the protective effects of AYB extract by comparing them with the protective effects of silymarin. Indeed, silymarin is well-known for its protective properties against various hepatic diseases and toxicities [30]. AYB extract rescued hepatocytes from APAP-induced hepatotoxicity to a similar extent as silymarin. AYB extract also prevented the induction of apoptosis and oxidative stress caused by APAP treatment, similar to the effects of silymarin.

The current study has some limitations that should be noted: first, the contents of active compounds in plant extracts depends on various factors, including the growth and harvest times, the climate of the cultivation area, and soil conditions. Therefore, further research should be conducted using AYB extracts from different regions to confirm the overall effect of AYB extract on APAP-induced liver toxicity. Second, in this study, we measured the diosgenin content, which is likely involved in the protective effects of AYB extract against APAP-induced hepatotoxicity. Therefore, future studies should compare the effects of diosgenin and the AYB extract to determine whether the hepatoprotective effects of AYB extract primarily depend on this component, thereby demonstrating the importance of using the extract over a single compound. Third, this study used the TIB-73 cell to evaluate the effect of AYB extract on APAP-induced hepatotoxicity. Therefore, future study should also be evaluated using other hepatic cells, such as HepG2 cells and primary hepatic cells.

## 5. Conclusions

The present study demonstrates that AYB extract exerts hepatoprotective effects against APAP-induced hepatotoxicity by promoting Nrf2 nuclear translocation, activating downstream antioxidant genes, inhibiting mitochondrial stress and dysfunction, and alleviating intracellular oxidative and ER stress. Therefore, AYB extract could be used to develop therapeutic strategies to mitigate or protect against APAP-induced hepatotoxicity.

## Data Availability

The data presented in this study are available on request from the corresponding author.

## Supplementary Materials

The following supporting information can be downloaded at: https://www.mdpi.com/article/10.3390/nu17060966/s1, Table S1: List of antibodies; Table S2: Specific primer sequences for qRT-PCR.
