# Supplementary material for: Aerial Yam Bulbils Protect Against APAP-Induced Hepatotoxicity by Inhibiting Oxidative Stress and Mitochondrial Dysfunction Through Nrf2 Activation
Source: Nutrients. 2025 Mar 10;17(6):966. doi: 10.3390/nu17060966 (PMC11944312; doi:10.3390/nu17060966)
Supplement: Supplementary file 1 [file nutrients-17-00966-s001.zip › nutrients-3495772-supplementary.pdf]

**Supplementary Table S1.** List of antibodies

| Antibody | Catalog number | Working dilution | Company |
|---|---|---|---|
| B-cell lymphoma 2 (Bcl-2) | sc-492 | 1:1,000 | Santa Cruz Biotechnology |
| Bcl-2-associated X protein (Bax) | #2772 | 1:1,000 | Cell signaling |
| pro-caspase 3 | #9665 | 1:1,000 | Cell Signaling |
| Cleaved caspase 3 | #9664 | 1:1,000 | Cell Signaling |
| catalase | #14097 | 1:1,000 | Cell signaling |
| Glutathione peroxidase (GPx) | #1341 | 1:1,000 | Cell signaling |
| superoxide dismutase 1 (SOD1) | sc-101523 | 1:1,000 | Santa Cruz Biotechnology |
| superoxide dismutase 2 (SOD2) | #13141 | 1:1,000 | Cell signaling |
| phosphorylated extracellular signal-regulated kinase 1/2 (ERK 1/2) | #9101 | 1:1,000 | Cell signaling |
| ERK 1/2 | #9102 | 1:1,000 | Cell signaling |
| Phosphorylated c-Jun N-terminal kinase (p-JNK) | #3251 | 1:1,000 | Cell signaling |
| JNK | #9252 | 1:1,000 | Cell signaling |
| Phosphorylated p38 (p-p38) | #9211 | 1:1,000 | Cell signaling |
| P38 | #9212 | 1:1,000 | Cell signaling |
| phosphorylated pancreatic ER kinase (p-PERK) | ab192591 | 1:1,000 | Abcam |
| PERK | #3192 | 1:1,000 | Cell signaling |
| phosphorylated eukaryotic translation initiation factor 2$\alpha$ (p-eIF2$\alpha$) | #3597 | 1:1,000 | Cell signaling |
| eIF2$\alpha$ | #9722 | 1:1,000 | Cell signaling |
| activating transcription factor 4 (ATF4) | #11815 | 1:1,000 | Cell signaling |
| C/EBP homologous protein (CHOP) | #2895 | 1:1,000 | Cell signaling |
| GADD45$\alpha$ | #4632 | 1:1,000 | Cell signaling |
| nuclear factor-erythroid-derived 2-like 2 (Nrf2) | sc-365949 | 1:1,000 | Santa Cruz Biotechnology |
| $\beta$-actin | sc-47778 | 1:1,000 | Santa Cruz Biotechnology |
| Lamin B1 | Sc-374015 | 1:1,000 | Santa Cruz Biotechnology |

**Supplementary Table S2.** Specific primer sequences for qRT-PCR

| Genes | Accession No. | Primers | |
|---|---|---|---|
| *PPARα* | NM_013196 | forward | 5′-TGAGCCCGGATATTGTAGCTGA-3′ |
| | | reverse | 5′-GCCGAATAGTTCGCCGAAAG-3′ |
| *NRF-1* | NM_001100708 | forward | 5′-CACTCTGGCTGAAGCCACCTTAC-3′ |
| | | reverse | 5′-TCACGGCTTTGCTGATGGTC-3′ |
| *ERRα* | NM_001008511 | forward | 5′-GGCAATGCACTGAACATCGAG-3′ |
| | | reverse | 5′-TGCTCCACAGCCTCAGCAT-3′ |
| *PGC1-α* | NM_031347 | forward | 5′-ACCGTAAATCTGCGGGATGA-3′ |
| | | reverse | 5′-AGTTTCATTCGACCTGCGTAAAGTA-3′ |
| *GCLC* | NM_012815.2 | forward | 5′-GTGGACACCCGATGCAGTA-3′ |
| | | reverse | 5′-CTTGTAGTCAGGATGGTTTGCAATA-3′ |
| *HMOX1* | NM_012580 | forward | 5′-ATTTGTCCGAGGCCTTGAA-3′ |
| | | reverse | 5′-CCAGGGCCGTATAGATATGGTA-3′ |
| *NQO1* | NM_017000 | forward | 5′-TGAGCCCGGATATTGTAGCTGA-3′ |
| | | reverse | 5′-GCATACGTGTAGGCGAATCCTG-3′ |
| *18S rRNA* | M11188 | forward | 5′-TGCGGAAGGATCATTAACGGA -3′ |
| | | reverse | 5′-CAGCCACCCGAGATTGAGCA -3′ |

PPAR$\alpha$; peroxisome proliferator activated receptor $\alpha$, NRF-1; nuclear respiratory factor-1, ERR$\alpha$; estrogen related receptor $\alpha$, PGC1-$\alpha$; PPARG coactivator 1$\alpha$, GCLC; glutamate-cysteine ligase, catalytic subunit, HMOX1; heme oxygenase 1, NQO1; NAD(P)H dehydrogenase, quinone, 18S; 18S ribosomal RNA
